# miR-134 inhibits chondrogenic differentiation of bone marrow mesenchymal stem cells by targetting SMAD6

**DOI:** 10.1042/BSR20180921

**Published:** 2019-01-30

**Authors:** Shaogang Xu, Xuejian Wu

**Affiliations:** Department of Orthopedics, The First Affiliated Hospital of Zhengzhou University, No. 1 Jianshe East Road, Zhengzhou City, Henan Province, China

**Keywords:** Bone marrow mesenchymal stem cells, Chondrogenic differentiation, miR-134, SMAD6

## Abstract

Various miRNAs have been reported to regulate the chondrogenic differentiation of bone marrow mesenchymal stem cells (BMSCs); however, whether miR-134 plays a role in this biological process remains undetermined. In the present study, we first evaluated the chondrogenic differentiation of BMSCs by Alcian blue staining, and examined the miR-134 expression by quantitative real-time PCR (qRT-PCR) during this process. And miR-134 inhibitor was used to investigate the functions of miR-134 in chondrogenic differentiation of BMSCs by Alcian blue staining, qRT-PCR, and Western blot. Subsequently, the correlation between miR-134 and SMAD6 was assessed via bioinformatics analysis and dual-luciferase reporter assay. Finally, the role of SMAD6 in chondrogenic differentiation of BMSCs was also determined through Alcian blue staining, qRT-PCR, and Western blot. As results showed that miR-134 expression was significantly down-regulated during chondrogenic differentiation, and inhibition of miR-134 obviously promoted chondrogenic differentiation. Dual-luciferase reporter assay indicated that miR-134 could directly target the 3′-UTRs of SMAD6, inhibit miR-134 expression in BMSCs, and up-regulate SMAD6 expression. Moreover, we found that overexpression of SMAD6 significantly promoted chondrogenic differentiation, and that SMAD6-induced promotion of chondrogenic differentiation could be reversed by miR-134 mimics. In conclusion, our findings suggest that miR-134 may act as a negative regulator during chondrogenic differentiation of BMSCs by interacting with SMAD6.

## Introduction

The damage to articular cartilage, primarily caused by trauma and excessive mechanical conditions, is the most common clinical disease [1,2]. Articular cartilage damage in severe cases probably leads to osteoarthritis (OA), which subsequently causes disability and chronic pain, producing a significant social and economic burden in the world [3–5]. It was reported that the economic costs of OA in the United States were estimated at 15 billion dollars in 2011 alone, making OA the second most expensive disease [6]. The conservative interventions of OA mainly include injection of intra-articular hyaluronic acid, and oral non-steroidal anti-inflammatory drugs; however, these treatments could only alleviate chronic pain [7–9]. Although surgical arthroplasty is applied to treat severe degenerative joint diseases, it may cause several severe postoperative complications [10,11]. Therefore, it is essential to explore some new effective therapeutic measures for articular cartilage damage.

Bone marrow mesenchymal stem cells (BMSCs) are one of the mesenchymal stem cells that can be isolated and differentiated into multiple cell types *in vitro*, such as osteoblasts, adipocytes, and chondrocytes [12,13]. Due to chondrocytes’ participation in the formation of cartilage tissues, chondrogenic differentiation of BMSCs may play an important role in the regeneration of cartilage [14–16]. Therefore, BMSC transplantation is currently considered to be the most promising measure for OA therapy [17,18]. A variety of growth factors have shown the ability to induce chondrogenic differentiation; however, the application of growth factors may contribute to calcification, limiting their clinical use [19,20]. MiRNAs are endogenous and evolutionary conserved non-coding RNA molecules with 20–24 nucleotides [21,22]. Evidences have demonstrated that miRNAs can regulate the expressions of target genes by directly binding to 3′-UTRs of their mRNA [23,24]. Since the first description of miRNAs in 1993, numerous miRNAs have been identified, and functional assays showed that miRNAs may be involved in almost whole biological processes, including cell proliferation, differentiation, and tumorigenesis [25,26]. Recently, increasing studies have revealed that miRNAs may participate in the chondrogenic differentiation of BMSCs, implying that regulation of miRNAs may be a new strategy for chondrogenic differentiation inducement [27–29].

In the present study, we aimed to investigate the miR-134 roles during chondrogenic differentiation of BMSCs, and attempted to reveal the underlying mechanisms. Due to the bioinformatics analysis results showing that SMAD6 is a targetted gene of miR-134, we hypothesized that the effects of miR-134 on chondrogenic differentiation of BMSCs may be by targetting SMAD6. Therefore, we tested this hypothesis in the present study and demonstrated that miR-134 may act as a negative regulator during chondrogenic differentiation of BMSCs by targetting SMAD6.

## Materials and methods

### Cell culture and chondrogenic differentiation

BMSCs of Sprague–Dawley rat (catalog number RASMX-01001) were provided by Cyagen Biosciences, Inc. (Guangzhou, China) and cultured at 37°C with the Dulbecco’s modified Eagle’s medium (DMEM; Gibco, MD, U.S.A.), supplemented with 10% FBS, 10 mg/ml of streptomycin, and 10 U/ml of penicillin, in a 5% CO_2_ humidified incubator. For the chondrogenic differentiation of BMSCs, a Chondrogenic Differentiation Kit (Cyagen Bioscience, Inc., Santa Clara, CA, U.S.A.) was applied under the instruction provided by manufacturers. Briefly, BMSCs were cultured in modified DMEM medium, which supplemented with dexamethasone, ascorbate, sodium pyruvate, and transforming growth factor-β 3 (TGF-β), and the chondrogenic differentiation medium was refreshed every 2 days.

### Alcian blue staining

After being cultured in normal DMEM or chondrogenic medium for 0, 14, and 21 days, BMSCs were fixed using 4% formalin for 30 min at 4°C. After being washed with PBS for 5 min, three times, BMSCs were then incubated with 1% Alcian blue 8GX (Sigma) at room temperature for 1 h. Subsequently, stained BMSCs were washed with deionized water for three times, and signals were detected by a flatbed scanner (HP, Palo Alto, CA, U.S.A.). Image-Pro Plus software (Media Cybernetics, Inc., Bethesda, MD) was applied to quantitate the staining intensity of cells.

### RNA extraction and quantitative real-time PCR assay

Total RNAs of culture BMSCs at 0, 14, and 21 days were extracted by an RNAiso Plus reagent (Takara Biotechnology, Japan) according to the protocols obtained from the manufacturer and the RNA concentration was determined using a spectrophotometer at 260 nm, and 2 μg of total RNAs were reverse transcribed into cDNA using BestarTM qPCR RT kit (#2220, DBI Bioscience, China). The qPCR reaction was performed using a BestarTM qPCR MasterMix kit (DBI Bioscience, China) with the Applied Biosystems 7500 PCR system (Applied Biosystems; Thermo Fisher Scientific, Inc.). The sequence of primers used in the present study is shown in Table 1. All primers were synthesized by Sangon, Shanghai, China. The miR-134 expression was normalized to U6. Collage II, SOX9, aggrecan, and SMAD6 expressions were normalized to GAPDH.

**Table 1:** Primer sequences for quantitative real-time PCR analysis

ID	Sequence (5′- 3′)
GAPDH F	CCTCGTCTCATAGACAAGATGGT
GAPDH R	GGGTAGAGTCATACTGGAACATG
Sox9 F	CCTAACGCCATCTTCAAGGC
Sox9 R	TTGCACGTCTGTTTTGGGAG
Aggrecan F	CTGTTATCGCCACTTTCCCG
Aggrecan R	CCCCTCTCATGCCAGATCAT
Col II F	GCCAGGATGCCCGAAAATTA
Col II R	CGTCAAATCCTCCAGCCATC
SMAD6 F	TTGCAACCCCTACCACTTCA
SMAD6 R	TTGGTGGCATCTGGAGACAT
U6 F	CTCGCTTCGGCAGCACA
U6 R	AACGCTTCACGAATTTGCGT
All R	CTCAACTGGTGTCGTGGA
rno-miR -134-5p	TGTGACTGGTTGACCAGAGGGG
rno-miR-134-5p F	ACACTCCAGCTGGGTGTGACTGGTTGACCA

### Cell transfection

Both miR-134 mimics and inhibitors were purchased from GenePharma (Shanghai, China). Before transfection, cells were seeded in 24-well plates and cultured at 37°C for 24 h, then 50 nM miR-134 mimics or inhibitors were added into the medium with lipofectamine 2000 (Invitrogen) and cultured for another 24 h. Nonsense control (NC) of miR-134 was used as control and transfected under the same condition.

### Western blot assay

For protein extraction, the cultured BMSCs were lysed with RIPA buffer containing PMSF, and then cell lysis was centrifuged at 10000 ***g*** for 15 min at a low temperature. After collecting the total protein extracts, a BCA Protein Assay Kit (Beyotime, China) was used to quantitate the protein concentration. Each sample (40 μg) of protein was loaded and separated by 10% SDS-PAGE, and then transferred onto PVDF membranes (Millipore, U.S.A.). Subsequently, membranes were incubated with 5% non-fat milk in TBST at room temperature for 2 h to block non-specific sites. The membranes were then incubated with corresponding primary antibodies against collage II (1:5000, Rabbit, ab34712, Abcam), SOX9 (1:3000, Rabbit, ab185230, Abcam), aggrecan (1:1000, Rabbit, ab36861, Abcam), and SMAD6 (1:500, Rabbit, ab80049, Abcam) overnight at 4°C. After being washed with TBST for three times, the membranes were subjected to incubation with HRP-conjugated donkey-anti-rabbit IgG (1:10000, ab6802, Abcam) for 2 h. Finally, the target blots were visualized by an enhanced chemiluminescence reagent, and protein expressions of collage II, SOX9, aggrecan, and SMAD6 were normalized to GAPDH (1:10000, Rabbit, ab8245, Abcam).

### Plasmid construction and dual-luciferase activity assay

The 3′-UTRs of SMAD6 including the target binding sites for miR-134 were chemically synthesized and cloned into pcDNA3.0 vector (Invitrogen) to form pcDNA3-SMAD6-WT plasmid, and the mutant SMAD6 binding sites were also cloned into pcDNA3.0 to produce pcDNA3-SMAD6-Mut plasmid. For the luciferase reporter assay, BMSCs (2 × 10^5^ cells/well) were cultured in 24-well plates for 24 h, then 100 ng of pcDNA3-SMAD6-WT or pcDNA3-SMAD6-Mut plasmids were co-transfected into BMSCs cells with 100 pmol of miR-134 mimics or NC using Lipofectamine 2000 (Invitrogen) following the protocols obtained from manufacturers. The luciferase activity was determined by a Dual Luciferase Reporter Assay System (Promega), and firefly luciferase activity was normalized to Renilla luciferase activity.

### Statistical analysis

Data of the present study were all expressed as mean ± SEM, and statistical analysis was performed by the Graphpad software (Ver. 7.0, U.S.A.). One-way analysis of variance was applied to assess the difference between means, and the difference between means was considered significant if *P*<0.05.

## Results

### miR-134 expression was down-regulated during chondrogenic differentiation of BMSCs

**Figure 1:**
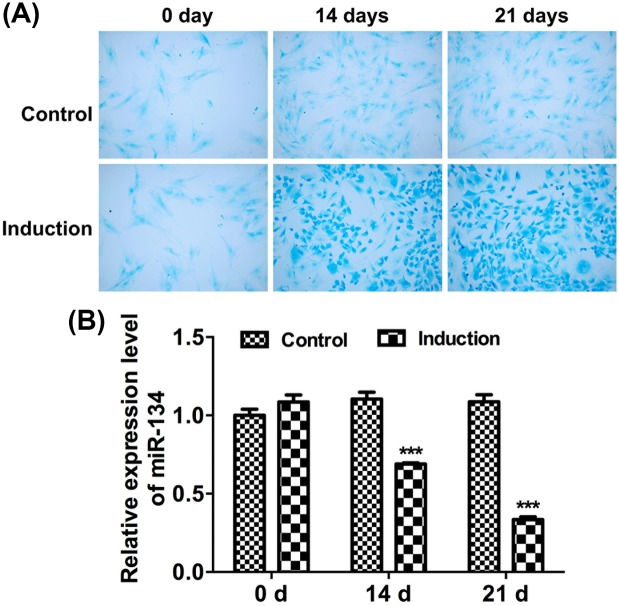
miR-134 expression in BMSCs during chondrogenic differentiation (**A**) Alcian blue staining of BMSCs cultured in control or chondrogenic medium at 0, 14, and 21 days (10× magnification). (**B**) The relative mRNA expression was determined by qRT-PCR in BMSCs cultured in control or chondrogenic medium at 0, 14, and 21 days (^***^P<0.001).

Since glycosaminoglycan deposition is a critical indicator of cartilage extracellular matrix accumulation, Alcian blue staining was performed to assess chondrogenic differentiation of BMSCs by examining glycosaminoglycan accumulation at 0, 14, and 21 days. As results indicated that staining intensity of Alcian blue was significantly increased at 14 and 21 days under chondrogenic medium (induction) (Figure 1A). To determine whether miR-134 plays a role in the chondrogenic differentiation of BMSCs, quantitative real-time PCR (qRT-PCR) assay was applied at 0, 14, and 21 days of chondrogenic differentiation to examine miR-134 expression. Results showed that the relative expression level of miR-134 was remarkably down-regulated during chondrogenic differentiation of BMSCs (Figure 1B).

### Inhibition of miR-134 promoted chondrogenic differentiation of BMSCs

**Figure 2:**
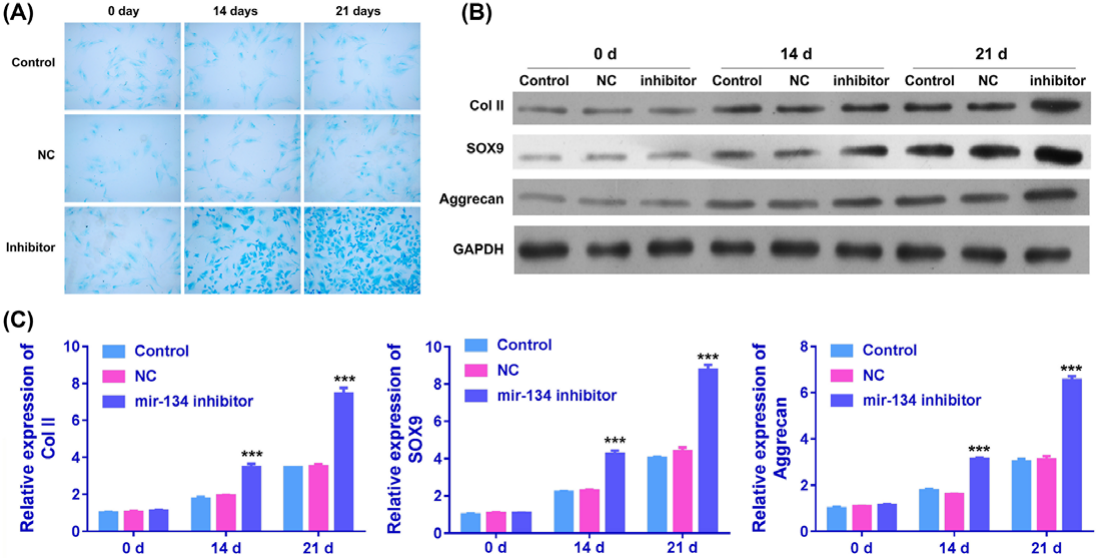
Effects of miR-134 inhibitor on chondrogenic differentiation of BMSCs (**A**) Chondrogenic differentiation of BMSCs treated with nothing, NC of miR-134, and miR-134 inhibitor was detected by Alcian blue staining. (**B**) The protein expressions of collage II, SOX9, and aggrecan were assessed with Western blot assay in control, NC, and inhibitor groups at 0, 14, and 21 days. (**C**) The relative mRNA expression levels of collage II, SOX9, and aggrecan were measured by qRT-PCR in miR-134 NC or inhibitor treated BMSCs at 0, 14, and 21 days (^***^P<0.001).

Inhibitor of miR-134 was transfected into BMSCs to evaluate whether miR-134 exhibits effects on chondrogenic differentiation. After treatment, BMSCs at 0, 14, and 21 days were stained with Alcian blue, and expressions of three chondrogenic differentiation markers: collage II, SOX9, and aggrecan were measured by qRT-PCR and Western blot assay. Results from Alcian blue staining indicated that staining intensity of BMSCs with miR-134 inhibitor transfection was obviously enhanced compared with control group and NC-treated group at 14 and 21 days (Figure 2A). Additionally, qRT-PCR and Western blot results suggested that BMSCs with miR-134 transfection significantly up-regulated the mRNA and protein expressions of collage II, SOX9, and aggrecan at 21 days (Figure 2B,C).

### miR-134 directly targetted the 3′-UTRs of SMAD6

**Figure 3:**
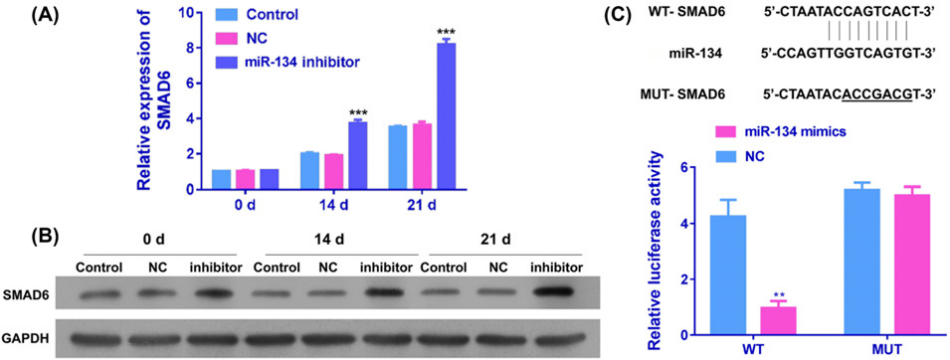
SMAD6 expression and correlation with miR-134 The relative mRNA (**A**) and protein (**B**) expressions of SMAD6 were measured by qRT-PCR and Western blot, respectively, in miR-134 NC or inhibitor-treated BMSCs at 0, 14, and 21 days (^***^P<0.001). (**C**) Upper panel, the complementary site sequence of miR-134, SMAD6, and mutant SMAD6. Lower panel, luciferase activity was detected in BMSCs co-transfected with pcDNA3-SMAD6-WT or pcDNA3-SMAD6-Mut and miR-134 mimics or NC.

To explore the underlying mechanisms of miR-134 involved in the chondrogenic differentiation of BMSCs, bioinformatics analysis was applied to screen the targets of miR-134. According to the prediction results from Targetscan software, miR-134 was shown to have binding sites in the 3′-UTRs of SMAD6 (Figure 3C, upper panel). To further identify whether miR-134 directly targets SMAD6, luciferase reporter vectors were established with wild-type SMAD6 3′-UTRs (pcDNA3-SMAD6-WT) and mutated SMAD6 3′-UTRs (pcDNA3-SMAD6-Mut). In the luciferase report assay, when pcDNA3-SMAD6-WT and miR-134 mimics were co-transfected into BMSCs, the luciferase activity was significantly attenuated compared with miR-134 NC (Figure 3C, lower panel). Nevertheless, co-transfection of pcDNA3-SMAD6-Mut and miR-134 mimics or NC had no obvious influence on the luciferase activity (Figure 3C, lower panel). In addition, we evaluated the effects of miR-134 inhibition on mRNA and protein expression of SMAD6 in BMSCs by qRT-PCR and Western blot, respectively. As results indicated that SMAD6 mRNA and protein expression in BMSCs transfected with miR-134 inhibitor were significantly increased at day 21 (Figure 3A,B).

### miR-134 inhibited chondrogenic differentiation of BMSCs via SMAD6

**Figure 4:**
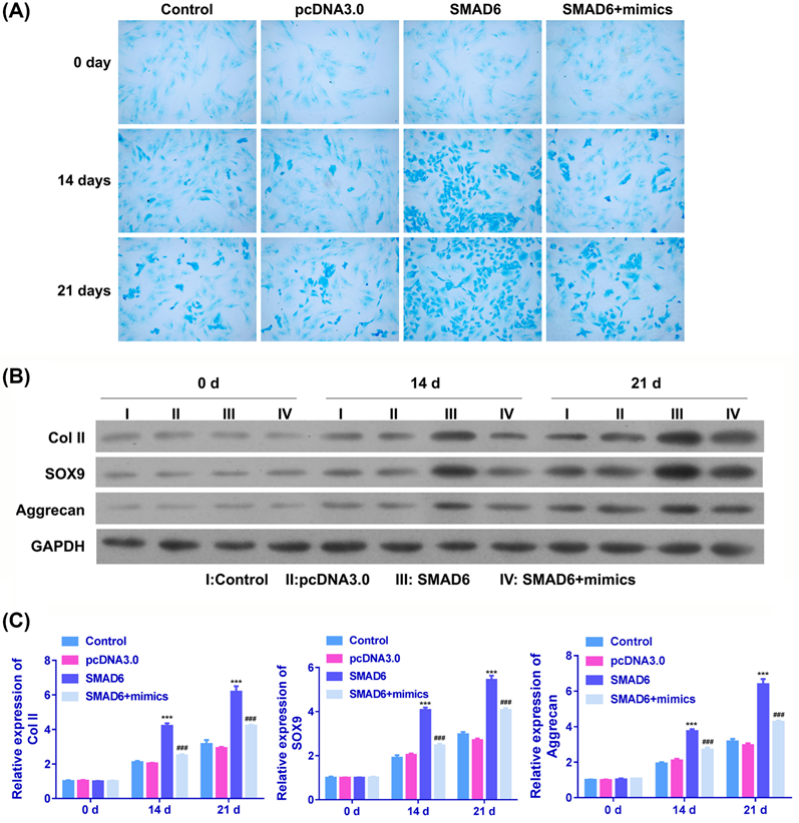
Effects of SMAD6 on chondrogenic differentiation of BMSCs (**A**) Chondrogenic differentiation of BMSCs treated with nothing, pcDNA3.0 vector, SMAD6, and SMAD6 plus miR-134 mimics was imaged at 0, 14, and 21 days. (**B**) Western blot analysis of collage II, SOX9, and aggrecan protein expressions in BMSCs treated with nothing, pcDNA3.0 vector, SMAD6, and SMAD6 plus miR-134 mimics. (**C**) The qRT-PCR analysis of collage II, SOX9, and aggrecan mRNA expressions in BMSCs treated with nothing, pcDNA3.0 vector, SMAD6, and SMAD6 plus miR-134 mimics (^***^P<0.001 compared with control, ^###^*P*<0.001 compared with SMAD6).

In order to further investigate the underlying mechanisms of interaction between miR-134 and SMAD6 during chondrogenic differentiation, BMSCs transfected with pcDNA 3.0 vector, SMAD6, and SMAD6 plus miR-134 mimics were subjected to Alcian blue staining analysis of chondrogenic differentiation, and qRT-PCR/Western blot analysis of collage II, SOX9, and aggrecan expressions. Alcian staining of BMSCs indicated that SMAD6 transfection remarkably increased the staining intensity compared with pcDNA3.0-transfected cells and blank control cells, and the application of miR-134 mimics could reverse the SMAD6-induced enhancement of intensity (Figure 4A). Moreover, results from qRT-PCR and Western blot indicated that collage II, SOX9, and aggrecan mRNA and protein expressions were significantly up-regulated in SMAD6-treated BMSCs compared with blank control cells and pcDNA3.0 vector-treated cells; however, the SMAD6-induced up-regulation of collage II, SOX9, and aggrecan could be abolished by miR-134 mimics (Figure 4B,C).

## Discussion

Cartilage injuries may be caused by excessive loading on joint, inflammation, and foreign body intrusion [30,31]. Virtually, cartilage could not regenerate or self-heal upon damages or degeneration, because of its low cell density and poor proliferative ability [32,33]. Currently, cartilage tissue regeneration and engineering are two primary treatment methods for cartilage injuries, the former includes all *in vivo* measures that aim to produce new tissues *in situ*, while the latter is mainly based on *in vitro* methods that attempt to engineer neo-cartilage tissues, which can be subsequently implanted into the patients [34–36]. Due to the requirement of donor material and its invasive character, the application of cartilage tissue regeneration is extremely limited, and cartilage tissue engineering obtained quick development during the past decades [37,38]. BMSCs are multipotent cells that could differentiate into musculoskeletal lineages, including chondrocytes, osteoblasts, and myocytes, making BMSCs the most appropriate cell source for cartilage tissue engineering [39,40].

The chondrogenesis process of BMSCs *in vitro* can be directed by multiple stimulus, such as growth factors, cell–matrix interaction, and mechanical loads [41]. Recently, miRNAs were also demonstrated to be involved in the process of chondrogenesis by interacting with several targetted genes [42,43]. An investigation about miRNA expression profiles was performed by Yang et al. [44] in MSCs during chondrogenic differentiation, and results indicated that eight increased miRNAs and five decreased miRNAs. Moreover, miR-30a was revealed to be up-regulated during chondrogenic differentiation of BMSCs, and miR-30a can promote this process by inhibiting delta-like 4 expression [45]. In addition, miR-145 and miR-495 were reported to be down-regulated, and they can both inhibit chondrogenesis by interacting with the critical chondrogenic transcription factor, SOX9 [46,47]. In the present study, we found that miR-134 expression was significantly down-regulated during chondrogenesis, and this process can be promoted in BMSCs treated with miR-134 inhibitor.

SMADs, the critical mediator of canonical TGF-β signaling pathway, are proteins that are responsible for transducing the TGF-β signal into nucleus. Previous studies revealed that SMADs could regulate the corresponding gene expressions by interacting with transcription factors in the nucleus when TGF-β signaling is activated [48–50]. Dysfunctions of SMAD proteins were presented in numerous diseases, such as kidney, renal, and heart diseases [51–53]. Recently, miR-140 and miR-199 were reported to modulate the process of chondrogenesis by regulating the expressions of SMAD3 and SMAD1, respectively [54,55]. Based on the results from bioinformatics analysis and dual-luciferase reporter assay, we found that SMAD6 was a targetted gene for miR-134, and inhibition of miR-134 in BMSCs could up-regulate the SMAD6 expression. Additionally, transfection with SMAD6 obviously promoted chondrogenesis of BMSCs, and this promotion could be abolished by miR-134 mimics. To our best knowledge, this is the first evidence of miR-134/SMAD6 axis participating in the chondrogenesis of BMSCs.

In conclusion, we revealed that miR-134 may serve as an important negative regulator during chondrogenic differentiation of BMSCs by targetting SMAD6. Further studies are also needed to validate and explore the deeper mechanisms and functions of miR-134 during chondrogenic differentiation of BMSCs by SMAD6. For example, inhibition of miR-134 followed by knockdown of SMAD6 will be performed to explore whether miR-134 can inhibit chondrogenic differentiation of BMSCs via SMAD6.

## Abbreviations

BMSC: bone marrow mesenchymal stem cell
Col: collage
DMEM: Dulbecco’s modified Eagle’s medium
GAPDH: glyceraldehyde phosphate dehydrogenase
HRP: horseradish peroxidase
MSC: Mesenchymal stem cells
OA: osteoarthritis
qRT-PCR: quantitative real-time PCR
RIPA: Radio Immunoprecipitation Assay
TBST: Tris-Buffered Saline Tween
TGF-β: transforming growth factor-β
